# Acute purulent mediastinitis with sequential bilateral pleural empyema caused by neck trauma: A unique occurrence, a case report

**DOI:** 10.1016/j.ijscr.2019.10.065

**Published:** 2019-11-03

**Authors:** Abdoulhossein Davoodabadi, Hamidreza Entezari, Hamidreza Talari, Ebrahim Razi

**Affiliations:** a Departments of Surgery, Trauma Research Center, Kashan University of Medical Sciences, Kashan, Iran; b Departments of Radiology, Kashan University of Medical Sciences, Kashan, Iran; c Departments of Pulmonology, Kashan University of Medical Sciences, Kashan, Iran

**Keywords:** DNM, Descending necrotizing mediastinitis, ICU, intensive care unit, Acute mediastinitis, Descending necrotizing mediastinitis, Pneumomediastinum, Blunt neck trauma, Pericardial thickening

## Abstract

**Background:**

Descending necrotizing mediastinitis (DNM) due to blunt trauma is very rare form of mediastinitis that can rapidly progress to septicemia and multi organ failure, in spite of optimal surgical management.

**Case presentation:**

We report a 28-year-old patient, previously healthy man, with acute descending necrotizing mediastinitis and late sequential bilateral pleural empyema, caused by blunt neck trauma. Although after admission, the course of his illness was complicated with septic shock, homodynamic instability, renal and pulmonary insufficiency, however he survived with on time diagnosis, aggressive surgical intervention, appropriate antibiotics administration and optimal management in the intensive care unit (ICU).

**Conclusions:**

Descending necrotizing mediastinitis due to blunt neck trauma is a rare occurrence, but can lead to late sequential bilateral empyema even after 18 days. With early diagnosis, aggressive drainage and antibiotic therapy, the patient life can be saved.

The lesson we took from this case is that: 1- blunt neck trauma rarely leads to descending necrotizing mediastinitis and late sequential empyema (even after 18 days).

2- If the patient does not recover from sepsis, despite optimal surgical management, contra lateral empyema or pericarditis should be considered.

## Highlights

• The lesson we took from this case is that: 1- blunt neck trauma rarely leads to descending necrotizing mediastinitis and late sequential empyema (after 18 days).
• 2- If the patient does not recover from sepsis despite optimal surgical management, contra lateral empyema or pericarditis should be considered.

## 1 Background

Descending necrotizing mediastinitis(DNM), is a rare but life-threatening infection that leads to a mortality rate up to 40%, despite antibiotic therapy and surgical interventions [1]. Only a few Primary cases of DNM have been reported in the literature and DNM occurs usually secondary to esophageal perforation and odontogenic abscess [[1], [2], [3]].

To date, one case of DNM with sequential bilateral pleural empyema has been reported in the literature due to blunt neck trauma with somehow similarity [4]. We present a DNM case caused by blunt neck trauma that was treated successfully in our trauma center.

## 2 Case presentation

A 28-year-old, previously healthy man was referred from the ward of otolaryngology, to our thoracic surgery department with dysphagia, muffled voice and swelling at his right sub mandibular area, 3days after blunt neck trauma. He complained from, dyspnea, dysphagia and pain in his right sub mandibular area and neck following falling of heavy bag (50 kg cement pocket) on his posterior neck. On clinical examination, swelling and subcutaneous emphysema was seen on admission day. He had not any dizziness or visual disturbance and his consciousness was normal. The first day after accident he had not serious symptoms except a moderate pain and tenderness on his neck but the pain and tenderness were increased, 24 h latter.

He did not have a history of a mass in his neck, dental abscess, diabetes mellitus, alcohol consumption, smoking or drug abuse.

On Physical examination: he had a blood pressure of 80/60 mm Hg, a Pulse rate of 130 beats/min, axillaries temperature of 38.4 °C, Respiratory rate was 35 breaths/min with dyspnea.

Tenderness, edema, erythema and emphysema was seen in the anterior of neck, but it was more significant in the right angle of mandible and para tracheal rather than the left.

Breath sound was diminished in both lungs fields but significantly was decreased in the right lung. The remainder of the examination revealed no pathologic findings.

Direct laryngoscopy, flexible bronchoscopy and esophagoscopy were performed showing no particular lesions.

Laboratory tests showed an elevated white blood cell (WBC) = 30,600/μl, low Hemoglobin (10.6 g/dL) and was elevated platelet count = 587 000 mm^3^. BUN = 50 mg/dL, Cr = 2.2 mg/dL, Na = 140 mg/dL, K 4 mg/dl, BS = 110 mg/dL, Urinalysis was normal but urine volume was diminished. An arterial blood gas analyzed in ICU with o2 supplement was as follows: pH = 7.36, PaO2 = 80 mmHg, PaCO2 = 50 mmHg and SaO2 = 90%. The electrocardiogram was normal, HIV test also was negative. Empirical broad-spectrum intravenous antibiotic (imipenem 500 mg/8 h + Ciprofloxacin 400 mg/12 h iv. and Metronidazole 500 mg/8 h) were prescribed till the results of culture to be obtained.

**Fig. 1:**
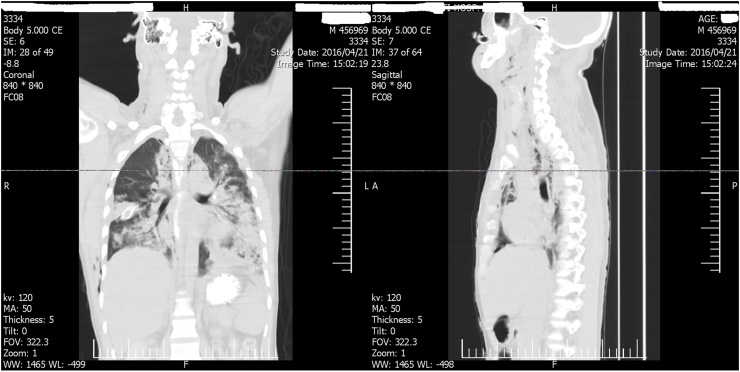
Coronal and sagital reformat view of chest show pneunomomediatinum and emphysema in cervical space.

**Fig. 2:**
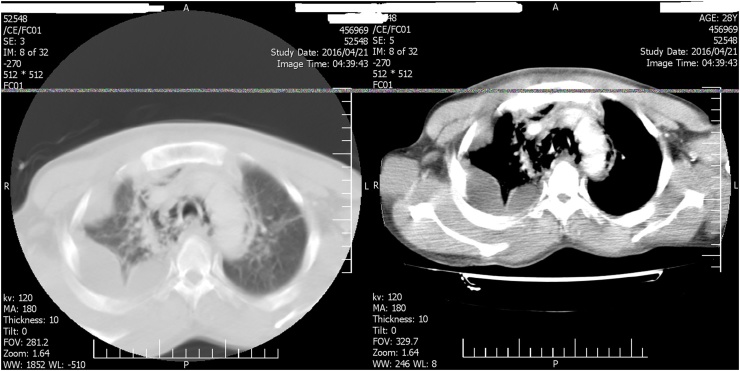
Axial lung and mediastinal window CT scan, show loculated pleural effusion and pleural enhancement due to empyema in right hemi thorax.

Coronal and sagittal reformat view of chest CT-scan of neck and thorax showed: pneunomomediastinum and emphysema in cervical space, extensive subcutaneous emphysema of the neck, around the right ramus of the mandible and right masseter muscle that extended into the left side of the neck and tracked down into the anterior mediastinum (Fig. 1). Computed tomography of thorax also revealed bilateral pleural effusion, more predominant at right side with loculated empyema. Cervical and dorsal vertebrae were intact (Fig. 2). The diagnosis of DNM was made on base of clinical findings, severe cervical and pharyngeal infection and radiologic features of mediastinitis on CT scan.

First, a 32 F chest tube was inserted in the right pleural cavity, approximately 500cc pus was drained and a sample for antibiogram was obtained. The patient was resuscitated and prepared for surgery after adequate urine output was achieved.

A right poster-lateral thoracotomy, through 5th intercostals space with endo tracheal general anesthesia was performed. During thoracotomy loculations and infected septa were disrupted and evacuated, the pleura of the upper esophagus was excised and the para esophageal planes opened until distal of esophagus. The mediastinum entirely was exposed, extensive debridement of necrotic tissue, irrigation of the paravertebral and para esophageal planes was done.

**Fig. 3:**
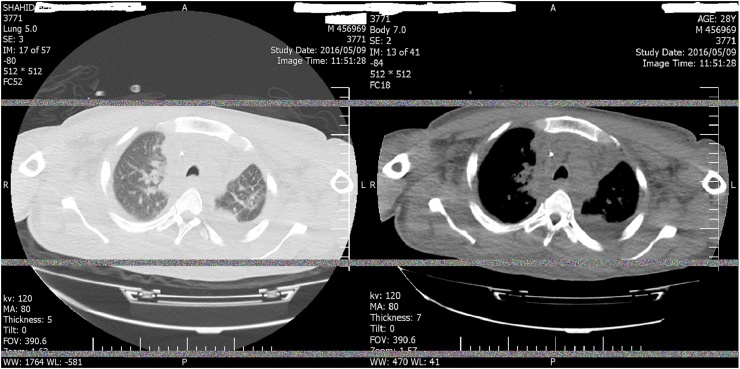
Axial lung and mediastinal window CT Scan, after 18 days show loculated empyema left hemi thorax with pericardial thickening (7 mm) after 18 day the first operation.

At the same time the neck was explored, copious amounts of pus and necrotic tissue in the submandibular triangle, parotid region, retro mandibular and pharyngeal spaces as much as possible was removed and irrigated with normal saline adequately, drainage was attained with three latex Penrose drains in different locations, then the patient was transferred to ICU with oral intubation. During18day of the post-operative period, the patient’s status despite of given high dose, antibiogram matched antibiotics and revealed signs of sepsis and pulmonary insufficiency. Bed side echocardiography showed pericardial thickening and effusion, repeated chest CT scan showed loculated empyema in left side with pericardial thickening (7 mm) without massive effusion or tamponade (Fig. 3). According to this finding, a left postero-lateral thoracotomy, through the 5th intercostal space was done and all of the loculations and infected spaces in pleura and mediastinum were evacuated.

Extensive debridement of the empyema and necrotic tissue was done in the same manner as on the right side, pericardia tap revealed serous fluid which was sent for culture. Recovery was relatively rapid after the 2th operation, the General condition improved rapidly, temperature became normal on the 4^th^ post-operative days and the patients was extubated on the 6^th^ post operative days.

The total duration of management was longed 29days. We also performed an upper gastrointestinal endoscopy but there were no pathological findings. Smear and culture from the pericardia tap were negative.

Culture of pleural fluid, revealed Poly microbial infection we did not perform a tracheotomy and weaned the patient from ventilator on 24^th^ admission day then liquid diet began. The patient had no complaints of hoarseness or dysphagia after extubation.

Before the patient’s discharge from the hospital, a control thoracic CT-scan showed relatively complete resolution of the pneumomediastinum and subcutaneous emphysema.

Outcome was favorable, during 90 days follow up, the pericardial thickening became normal and no evidence of tracheal stricture was seen. A written informed consent was obtained from the patient for possible publication including images for this case report.

SCARE compliance: this work has been reported in line with the SCARE criteria [5].

## 3 Discussion

DNM is a rare but potentially fatal complication of cervicofacial space infection, with a 64% mortality rate if combined with sepsis [3]. Early recognition with aggressive surgical and medical management, offers the best chance of survival [6]. In most case series, DNM, was reported in a mean age of the age 5-6 decade [7] with associated risk factors. Our patient was much younger, and completely healthy without any history of risk factors, disease, or addiction, he had not history of dental abscess, palpation of lumpy mass or indurations in his neck.

Majority of cases of acute mediastinitis are caused by esophageal rupture due to trauma, neoplasm, surgery, endoscopy and odontogenic abcess [8]. In our case the esophagus was normal and intact, in both barium swallow and esophagoscopy. There was also no any evidence of oropharyngeal and dental infection. He was a healthy worker, who developed cervicofacial space infection due to blunt neck trauma that eventually descended to the mediastinum and developed late sequential bilateral pleural empyema.

DNM due to blunt neck trauma, as this case, is a very rare and unique occurrence, only one case with some similarity has been reported [4], so blunt neck trauma can potentially have disastrous effects, which leads to DNM and sequential bilateral pleural empyema.

The diagnosis of DNM was made based on clinical finding of severe neck infection, radiologic features of mediastinitis on CT scan and necrotizing mediastinal infection at operation fields [9].

Falling of a heavy object (50 kg cement pocket) on the back and lateral neck may have acted as a traumatic contusion of the submandibular triangle, parotid region, oropharyngeal spaces and paraesophageal plane, causing disruption of tissue membrane and disintegrate of mucosal barriers and predisposing to microbial translocation from the oropharynx to the deep cervical fascia without macroscopic esophageal rupture.

The culture of organisms of pus and mediastinal necrotic tissue in our patient was mixed polymicrobial aerobic and anaerobic gas-producing bacteria, supporting the oropharyngeal origin. The etiological organisms of the meditational infections are mostly mixed polymicrobial aerobic and gas producing [10]. The effect of gravity and the negative intrathoracic and pleural pressure during inspiration, are important pathophysiological factors in the extension of deep neck infections to the mediastinum [11].

Presence of any coexisting morbidities such as diabetes mellitus, alcoholism, smoking, and drug abuse can further facilitate this rapid extension and increase the occurrence of complications. Although our patient had none of mentioned risk factors.

The surgical approach for optimizing drainage and debridement in DNM which extends to the lower anterior and posterior mediastinum and para esophageal plane, ideally is a standard posterolateral thoracotomy combine with cervical approach [11].

Inadequate surgical debridement and derange of infected pockets in the thoracic cavity, mediastinum and cervical plan, leads to prolonging ICU stay, deterioration of the general condition and causes new infectious foci. For this purpose, although the patient was fragile, we preferred staged standard posterolateral thoracotomy to video assisted thoracoscopy which was reported more tolerable and safe [13]. Standard posterolateral thoracotomy in DNM provides very good exposure to the pleural cavity, the pericardium and all mediastinal compartments. Posterolateral thoracotomy also provides the broadest exposure of the pre vertebral and para esophageal planes without the risk of sternal incision site infection [12]. However posterolateral thoracotomy, does not permit contralateral mediastinal debridement or exploration of the contralateral pleural cavity, therefore overcome to this drawback, the clamshell approach [14], median sternotomy, thoracoscopic approach and recently, video-assisted mediastinoscopy also were used [[15], [16], [17]].

In this case, surgical drainage and debridement of the neck, mediastinum and right hemi thorax was done adequately, but in spite of vast broad spectrum antibiotics, contra lateral pleural empyema and pericardial thickening was developed during18 days after the first operation and was led to continuing septic condition.

With contra lateral thoracotomy, the condition of the patient improved rapidly. So in any patient, who does not response to optimal treatment, requires early diagnosis and radiologic reevaluation. CT scan is indispensable because clinical examination alone is reliable in only 55% of cases [1,7,18].

Our patient had oral endotracheal tube during treatment with mechanical ventilation. Since opening the cervical fascia in tracheostomy exposes the patient to the contamination of pretracheal space and the risk of caudal spread of the infection to the mediastinum [6,11]. Opening of the airway by tracheostomy in DNM can result propagation of the infection to the lungs, So intubation was preferred to tracheostomy.

Familiarity of physicians with this rare but potentially serious complication due to blunt neck trauma even in previously healthy patients could prevent the delayed diagnosis and performing on time optimal management. Any delay in appropriated approach may leads to serious complications including septic shock, bilateral empyema prolonged mechanical ventilation and ICU stay.

## 4 Conclusion

With early diagnosis of DNM due to blunt neck, trauma, aggressive debridement, drainage, antibiotic therapy and adequate postoperative care can save the patient life. Inadequate surgical drainage in any possible thoracic cavity, mediastinum and cervical spaces can leads to deterioration of general condition and prolonged ICU stay, so well planned option for best drainage has more important role in final outcome.

Strengths and limitations of the study: we could successful treatment with on time diagnosis and careful clinical monitoring through the disease course in a rare case of Descending necrotizing mediastinitis due to blunt neck trauma. Esophagial assessment has some limitations during intubation period, so if possible pre intubation must to be done.

## Declaration of Competing Interest

The authors declare no conflicts of interest.

## Sources of funding

There is no source of funding other than the authors.

## Ethical approval

Due to Critical status and need urgent operation I ethical approval has been exempted by our institution.

## Consent

A written informed consent was obtained from the patient for possible publication accompanying images for this case report.

## Author contribution

All authors completed study concept, data collection, data analysis & interpretation, writing the paper.

## Registration of research studies

N/A.

## Guarantor

Abdolhossein Davoodabadi MD.

## Provenance and peer review

Not commissioned, externally peer-reviewed.
